# A Fire on the Hearth

**Published:** 1859-12

**Authors:** 


### A FIRE ON THE HEARTH.

How it carries us back to the days of happy childhood, when roaring wood fires blazed high up the chimney, with the shadows of flame flickering on the wall; pussy cat in one corner, the dog in the other, and the children sprawling about promiscuously, some reading, some tittering, some nodding, and all happy!

Ever since we came to New York, we have had "a longing and a sighing" for the leeks and onions of Egypt, the peculiar institution not excepted. We have, as to fires, made all sorts of experiments and changes, with portable furnaces, and unportable; grates, and no grates; ten plate stoves, and stoves with no plates at all, and at long last, we have gotten back to first principles, a fire on the hearth. Here, in our office, at 42 Irving Place, New York, we have the greatest uniquity in Gotham, a hard coal fire burning fiercely, flat on the hearth, on a level with the floor, with a great flaring jam, capacious enough to receive our four, from twelve, down to six, with the upper and the lower house, and "Samuel" and grandmother besides with room to spare for any welcome "prophet" who may chance to call, for the ever ready "chamber."

There is no visible "blower," very little dust, and absolutely no gas. The ashes need removing but once a year. By the extra heat, pure air direct from out doors, is warmed and conveyed into a room above, without the possibility of meeting with a red hot metallic surface, or with any corrupting source whatever, it is simply pure air warmed. To any one subscribing at our office for Hall's Journal of Health for 1860, we will exhibit this rare sight free of charge!! *All* are earnestly invited to "come and see." To those who dislike furnace heat, this open, low down, air tight, easily regulated grate, with its large broad bed of burning coals, will be a great desideratum, and yet it is the only one in New York.

